# Association between serum S100A11 levels and glucose metabolism in diabetic process

**DOI:** 10.1186/s13098-023-01004-1

**Published:** 2023-03-06

**Authors:** Yao Wu, Shaobo Wu, Fang Li, Ting Zeng, Xiaohe Luo

**Affiliations:** 1 Department of Laboratory Medicine, School of Medicine, Chongqing University Three Gorges Hospital, Chongqing University, No.165, Xincheng Avenue, Wanzhou District, Chongqing, 404000 China; 2 The Center of Clinical Research of Endocrinology and Metabolic Diseases in Chongqing, Chongqing University Three Gorges Hospital, Chongqing, 404100 China; 3 Department of Endocrinology, Chongqing University Three Gorges Hospital, Chongqing, 404100 China

**Keywords:** Diabetes mellitus, Impaired glucose tolerance, HbA1c, S100A11

## Abstract

**Background:**

Diabetes mellitus (DM) is a prevalent non-communicable metabolic disease, and S100A11 is a newly identified gene closely related to metabolism. The association of S100A11 with diabetes is unclear. This study aimed to assess the relationship between S100A11 and markers of glucose metabolism in patients with different glucose tolerance and gender.

**Methods:**

This study included 97 participants. Baseline data were obtained, and the serum levels of S100A11 and metabolic markers (glycated hemoglobin [HbA1c], insulin release test, and oral glucose tolerance test) were measured. Linear and nonlinear correlations between serum S100A11 levels and HOMA-IR, HOMA of β, HbA1c, insulin sensitivity index (ISI), corrected insulin response (CIR), and oral disposition index (DIo) were analyzed. The expression of S100A11 was also detected in mice.

**Results:**

Serum S100A11 levels increased in patients with impaired glucose tolerance (IGT) of both genders. S100A11 mRNA and protein expression increased in obese mice. There were nonlinear correlations between S10011 levels and CIR, FPI, HOMA-IR, whole-body ISI in the IGT group. S100A11 was nonlinearly correlated with HOMA-IR, hepatic ISI, FPG, FPI, and HbA1c in the DM group. In the male group, S100A11 was linearly correlated with HOMA-IR and nonlinearly correlated with DIo (derived from hepatic ISI) and HbA1c. In the female population, S100A11 was nonlinearly correlated with CIR.

**Conclusions:**

Serum S100A11 levels were highly expressed in patients with IGT and in the liver of obese mice. In addition, there were linear and nonlinear correlations between S100A11 and markers of glucose metabolism, demonstrating that S100A11 has a role in diabetes.

*Trial registration* ChiCTR1900026990

**Supplementary Information:**

The online version contains supplementary material available at 10.1186/s13098-023-01004-1.

## Introduction

Diabetes mellitus (DM) is a highly prevalent chronic non-communicable metabolic disease. The number of people with DM is expected to reach 700 million in 2045 [1]. DM is characterized by chronic hyperglycemia caused by impaired insulin secretion and action [2], potentially causing damage to various tissues, including the eyes, kidneys, heart, blood vessels, and nerves [2]. The risk factors for DM include immune dysfunction and obesity due to overeating and reduced physical activity [3]. Moreover, many genes encoding insulin receptors, glucokinases, HLA antigens, and mitochondrial proteins are implicated in DM [4, 5].

The gene encoding S100A11, a member of the calcium-binding S100 protein family with two EF-hand structural domains that bind calcium ions and undergo conformational changes [6], is expressed in the human skin, spleen, kidney, stomach, lung, heart, and liver. S100A11 plays a role in tumors, metabolic diseases, neurological diseases, and vascular calcification [7–11]. Fadista et al. [12] found a positive correlation between S100A11 and glycated hemoglobin (HbA1c) by genomic and transcriptomic analysis of human pancreatic islets from 89 donors. HbA1c levels provide a measure of glycemia over the lifespan of erythrocytes (approximately 120 days) and thus serve as a glycemic control marker [13, 14]. This positive correlation suggests that S100A11 has a role in DM. This study evaluated the correlation between S100A11 and markers of glucose metabolism in patients with different glucose tolerance.

## Materials and methods

### Patient samples

A total of 97 cases of health check-ups or volunteers from Chongqing University Three Gorges Hospital who visited the hospital from November 2018 to November 2019 were included in the study. Women during pregnancy, those with a history of tumor, patients with severe liver and kidney diseases, cardiovascular diseases, hematological diseases, patients with clear familial hyperlipidemia and hypertension, and patients undergoing steroid intervention therapy were excluded. Subjects were divided into three groups according to the results of the OGTT experiment: 33 cases in the normal glucose tolerance (NGT) group, 26 cases in the impaired glucose tolerance (IGT) group, and 38 cases in the diabetes mellitus (DM) group.

### Human experiments

The gender and age of the study subjects were collected, and general clinical indices such as height, weight, waist circumference and hip circumference were measured by professionals, and body mass index BMI = body mass (kg)/height (m^2^) and waist-to-hip ratio WHR = waist circumference/hip circumference were calculated. Subjects maintained normal diet and exercise three days before blood sampling, fasted 8 ~ 12 h the night before blood sampling, took venous blood on the same day on an empty stomach, and immediately after blood collection took 75 g of glucose orally followed by 30 min, 60 min and 120 min respectively, and measured HbA1c, fasting plasma glucose (FPG), fasting plasma insulin (FPI), postprandial glucose (30 min, 60 min, 120 min), and postprandial insulin (30 min, 60 min, 120 min). The steady-state model was used to assess the insulin resistance index HOMA-IR = FPG (mmol/L)*FPI (mU/L)/22.5, and islet β-cell function HOMA-β = 20*FPI (mU/L)/(FPG (mmol/L)-3.5) [15, 16]. Calculation of insulin sensitivity index ISI (the liver) = 22.5*18/(FPG (mmol/L)*FPI (mU/L), ISI (the body) = 10,000/$$\sqrt{((\mathrm{FPG}*\mathrm{FPI})*\mathrm{mean\,OGTT\,blood\,glucose\,concentration}*\mathrm{mean\,insulin\,concentration})}$$, corrected insulin response is a measure of glucose-stimulated insulin secretion at 30 min of OGTT and provides an estimation of beta-cell function and was calculated as: CIR (uU/mL mmol/L-1 mmol/L-1) = 100*0.5hPPI/(0.5hPPG*(0.5hPPG -3.89)) (0.5hPPG needs to be > 4.44 mmol/L and > FPG). DIo provides an estimate of beta cell function adjusted for insulin resistance and takes into account the degree of insulin sensitivity, since CIR is driven by both glucose and insulin sensitivity. DIo is calculated by multiplying CIR by ISI [17–19].

### Animal experiments

Ob/ob and db/db mice were purchased and HFD (high-fat diet) fed C57BL/6 mice for 12 weeks, then mice were executed and liver tissue was taken and stored in liquid nitrogen for subsequent experiments. The proteins were quantified using the BCA protein analysis kit and separated on 12% polyacrylamide gels and then transferred to 0.45um PVDF membranes. These membranes were incubated overnight at 4 °C in antibodies against S100A11 (1:10,000, Proteintech, Chicago, USA), β-actin (1:2000, ZSGB-BIO, Beijing, China), followed by horseradish peroxidase-labeled goat anti-mouse/rabbit immunoglobulin (1:20,000). Membranes were exposed to X-rays by incubation with ECL detection solution. Total tissue RNA was extracted using Trizol (Takara, Tokyo, Japan), and reverse transcription products of total sample RNA were prepared using a kit (Takara, Tokyo, Japan) and mRNA amplification was performed on a qTower (Analytik Jena AG, Jena, Germany) instrument. β-Actin was used as a standard control to normalize the relative mRNA expression of a specific gene via the delta-delta cycle threshold method. The primer used was: S100A11 (Forward primer GCGGGAAGGATGGAAACAACA, Reverse primer TCATCATGCGGTCAAGGACAC).

### Statistical analysis

Linear and nonlinear correlations between serum S100A11 levels and glucose metabolism indices were analyzed in different glucose tolerance and different gender populations, respectively. One-way ANOVA was used for normally distributed continuous variables, and Kruskal–Wallis’s test was used for skewed continuous variables, which were obtained by Fisher's exact probability test if the theoretical number of count variables was less than 10. Linear relationships between serum S100A11 and glucose metabolism indicators (assessed as FPG, FPI, 0.5hPPG, 0.5hPPI, HbA1c, HOMA-IR, HOMA-β, ISI, CIR, DIo) were analyzed using multivariate corrected linear regression and t-test. Regression coefficients and corresponding 95% confidence intervals (CI) were calculated. The adjusted models I and II were adjusted for different covariates according to different subgroups and different response variables.

We further investigated the nonlinear relationships between serum S100A11 and glucose metabolism indicators. Smoothing functions and segmented linear regression models were applied to adjust for glucose tolerance/gender, age, BMI, WHR, o.5hPPG/0.5hPPI/FPG/FPI/HbA1c. We used R packages (http://www.r-project.org) and Empower® (R) (www.empowerstats.com, X&Y Solutions Inc., Boston, MA, USA) for the statistical analyses.

## Results

### Patient characteristics

**Fig. 1:**
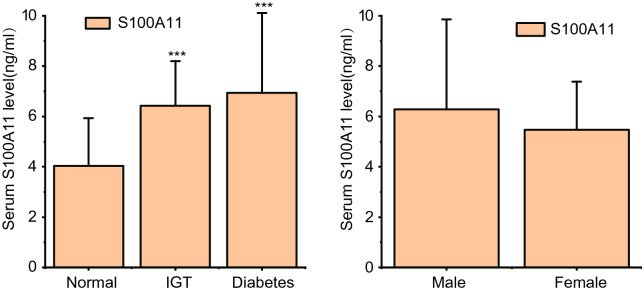
Normal patients vs impaired glucose tolerance and diabetes patients. ***P < 0.001 (K-W test). No difference between male and female groups

The study included 97 patients (56 females and 41 males) admitted to Chongqing University Three Gorges Hospital. Patient characteristics are shown in Table 1. The mean serum S100A11 level was 5.82 ng/mL. Serum S100A11 levels were significantly higher in the impaired glucose tolerance and diabetic groups than in the normal glucose tolerance group (P < 0.01). In addition, HbA1c, fasting plasma glucose (FPG), 0.5 h postprandial glucose (0.5 h PPG), 0.5 h postprandial insulin (0.5 h PPI), homeostasis model assessment of insulin resistance (HOMA-IR), HOMA of β-cell function (HOMA-β), insulin sensitivity index (ISI), corrected insulin response (CIR), and oral disposition index (DIo) were significantly different between these two groups (P < 0.05, P < 0.01). However, there were no significant differences in serum S100A11 levels between the genders (Fig. 1).

**Table 1:** Patient characteristics, stratified by glucose tolerance (N = 97)

Item	Total patients (N = 97)	Normal (N = 33)	IGT (N = 26)	Diabetes (N = 38)	P-value
S100A11(ng/mL)	5.82 (2.75)	4.04 (1.90)	6.43 (1.76)	6.94 (3.17)	< 0.001
Male	41 (42.27%)	13 (39.39%)	11 (42.31%)	17 (44.74%)	0.902
AGE (years)	49.77 (12.50)	47.48 (12.21)	50.58 (15.99)	51.21 (9.81)	0.323
BMI (kg/m^2^)	25.46 (3.93)	25.80 (4.00)	24.93 (2.79)	0.611	0.565
WHR	0.90 (0.07)	0.88 (0.07)	0.90 (0.08)	0.91 (0.06)	0.067
HbA1c (%)	6.88 (1.99)	5.73 (0.71)	6.01 (0.58)	8.47 (2.30)	< 0.001
FPG (mmol/L)	7.33 (3.03)	5.36 (0.45)	5.91 (0.56)	10.02 (3.34)	< 0.001
0.5hPPG (mmol/L)	12.28 (4.20)	8.97 (1.42)	10.89 (1.67)	16.11 (3.98)	< 0.001
FPI (uU/mL)	9.41 (5.69–15.87)	8.18 (6.39–12.03)	10.73 (6.77–18.59)	11.41(5.31–15.82)	0.515
0.5hPPI (uU/mL)	43.86 (22.35–74.47)	66.78 (41.98–92.69)	46.72 (36.27–75.53)	20.44(12.55–44.80)	< 0.001
HOMA-IR	2.86 (1.78–5.07)	1.94 (1.44–2.86)	2.77 (1.76–4.58)	4.07 (2.36–6.70)	0.013
HOMA-β	72.71 (32.66–111.90)	90.91 (70.17–122.79)	87.52 (53.64–137.19)	33.63 (22.02–69.04)	0.002
ISI (the liver)	6.30 (3.55–10.14)	9.28 (6.30–12.48)	6.55 (3.93–10.26)	4.43 (2.69–7.66)	0.002
ISI (the body)	0.55 (0.25–1.79)	0.36 (0.23–0.82)	0.42 (0.19–1.14)	1.03 (0.40–3.60)	0.041
CIR	58.12 (20.15–138.10)	141.56 (98.40–302.99)	73.42 (43.28–114.81)	14.81 (6.08–31.90)	< 0.001
DIo (the liver)	391.99 (102.55–1076.93)	1300.98 (911.61–2333.22)	443.40 (217.79–656.85)	56.50 (24.35–129.69)	< 0.001
DIo (the body)	26.57 (13.48–66.83)	66.83 (42.11–102.18)	31.65 (21.43–57.62)	14.12 (7.59–21.82)	< 0.001

Values are Mean (SD) or Median (Q1–Q3) or N (%). Groups were compared using the nonparametric Kruskal-Wallis (K-W) test and Fisher exact probability test

### Association between serum S100A11 and glucose metabolism markers

#### Linear association between S100A11 and glucose metabolism markers

Subgroup analysis by glucose tolerance and gender showed that serum S100A11 levels were negatively correlated with CIR in the IGT group in model 2 (Table 2). Nonetheless, the correlation was not significant in model 1 (Table 2), suggesting that FPG, FPI, and HbA1c mediated the effect of S100A11.

**Table 2:** Multivariate regression for effect of serum S100A11 level on metabolic markers in different models, stratified by glucose tolerance and gender

	CIR (β (95%CI)P)			HOMA-IR (β (95%CI)P)	
	Norma	IGT	Diabetes	Male	Female
Crude Mode l	29.66 (− 5.35, 64.67) 0.1069	− 11.39 (− 32.04, 9.25) 0.2902	0.04 (− 2.78, 2.87) 0.9756	0.48 (0.27, 0.69) < 0.0001	0.10 (− 0.45, 0.65) 0.7296
Adjusted Mode II	24.29 (− 11.19, 59.76) 0.1909	− 15.79 (− 33.98, 2.40) 0.1043	− 0.41 (− 3.42, 2.61) 0.7933	0.47 (0.26, 0.68) 0.0001	0.11 (− 0.36, 0.58) 0.6494
Adjusted Model II	21.57 (− 5.30, 48.45) 0.1287	− 17.32 (− 30.38, − 4.25) 0.0188	0.05 (− 2.19, 2.28) 0.9657	0.32 (0.06, 0.58) 0.0214	− 0.28 (− 0.77, 0.21) 0.2645

Crude model: we did not adjust other covariates

Adjusted model I: We adjusted gender, age, BMI, WHR (stratified by glucose tolerance). We adjusted age, BMI, WHR (stratified by gender)

Adjusted model II: We adjusted gender, age, BMI, WHR, HbA1c, FPG, FPI (stratified by glucose tolerance). We adjusted AGE, BMI, WHR, HbA1c, 0.5hPPI, 0.5hPPG, glucose tolerance (stratified by gender)

Subgroup analysis by gender showed that serum S100A11 levels were positively correlated with HOMA-IR in the male population after adjusting for covariates (Table 2). Moreover, there was a linear relationship between serum S100A11 levels and HbA1c, DIo, FPG, and 0.5-h PPG (Additional file 1: Table S1) in the male population after adjusting for age, BMI, and WHR. However, the correlation was not significant after adjusting for HbA1c, FPG, 0.5-h PPG, FPI, 0.5-h PPI, and glucose tolerance levels; thus, these indicators may have enhanced the correlation (Additional file 1: Table S1). The results were similar in the female population (Additional file 1: Table S2).

#### Nonlinear association between S100A11 and glucose metabolism markers

**Fig. 2:**
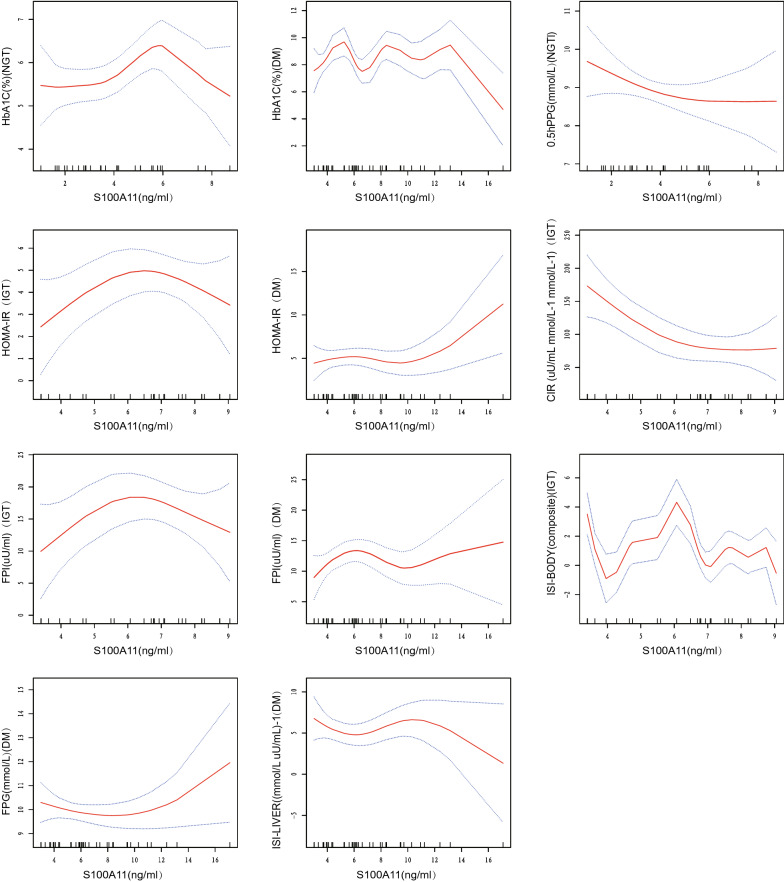
Multivariate adjusted smoothing spline plots of metabolic markers by serum S100A11, stratified by glucose tolerance. Red dotted lines represent the spline plots of S100A11 and blue dotted lines represent the 95% confidence intervals of the spline plots. Adjusted for Glucose tolerance, age, BMI, WHR, 0.5hPPG/0.5hPPI/HbA1c/FPG/FPI

Nonlinear relationships between serum S100A11 levels and metabolic markers were evaluated by curve-fitting, and a piecewise linear regression model was constructed (Fig. 2). In the normal population, serum S100A11 levels were nonlinearly correlated with HbA1c and 0.5-h PPG after multivariate adjustment. Serum S100A11 levels lower than 4.19 ng/mL were negatively correlated with 0.5-h PPG (a decrease of 0.51 mmol/L per 1 ng/mL increase in S100A11). S100A11 levels higher than 4.19 ng/mL were positively but weakly correlated with 0.5-h PPG. Serum S100A11 levels lower than 5.78 ng/mL were positively correlated with HbA1c (an increase of 0.26% per 1 ng/mL increase in S100A11). Serum SS100A11 levels higher than 5.78 ng/mL were negatively but weakly correlated with HbA1c (Table 3).

**Table 3:** Threshold effect analysis of S100A11 on metabolic markers by using two-piecewise linear regression, stratified by glucose tolerance

	Inflection points of HbA1c			Inflection points of FPG			Inflection points of 0.5hPPG			Inflection points of FPI		
	β	(95%CI)	P	β	(95%CI)	P	β	(95%CI)	P	β	(95%CI)	P
Normal	> 5.78	0.26 (0.04, 0.47)	0.0316	> 4.19	0.16 (− 0.01, 0.32)	0.0741	> 4.19	− 0.51 (− 0.98, − 0.03)	0.0482	> 2.76	2.43 (− 4.47, 9.33)	0.4965
	≤ 5.78	− 0.43 (− 0.85, − 0.02)	0.0538	≤ 4.19	− 0.06 (− 0.20, 0.08)	0.4184	≤ 4.19	0.15 (− 0.26, 0.55)	0.4804	≤ 2.76	− 0.71 (− 2.69, 1.27)	0.4906
IGT	> 8.74	− 0.11 (− 0.22, 0.01)	0.1010	> 8.29	0.06 (− 0.08, 0.21)	0.3874	> 3.63	− 10.72 (− 23.94, 2.50)	0.1329	> 6.48	4.07 (0.56, 7.58)	0.0383
	≤ 8.74	3.41 (− 0.03, 6.85)	0.0708	≤ 8.29	0.06 (− 0.08, 0.21)	0.3874	≤ 3.63	0.40 (− 0.05, 0.85)	0.1028	≤ 6.48	− 4.29 (− 8.85, 0.28)	0.0856
Diabetes	> 12.4	0.11 (− 0.05, 0.28)	0.1979	> 12.4	− 0.09 (− 0.26, 0.08)	0.3082	> 3.97	2.36 (− 1.82, 6.55)	0.2783	> 4.1	8.67 (1.79, 15.56)	0.0201
	≤ 12.4	− 0.90 (− 1.63, − 0.18)	0.0213	≤ 12.4	0.88 (0.15, 1.62)	0.0262	≤ 3.97	− 0.10 (− 0.39, 0.20)	0.5325	≤ 4.1	− 0.36 (− 0.97, 0.25)	0.2588

	Inflection points of HOMA-IR			Inflection points of ISI (the liver)			Inflection points of ISI (the body)			Inflection points of CIR		
	β	(95%CI)	P	β	(95%CI)	P	β	(95%CI)	P	β	(95%CI)	P
Normal	> 2.76	0.88 (− 0.66, 2.42)	0.2750	> 4.19	− 1.06 (− 4.13, 2.01)	0.5061	> 5.08	− 0.30 (− 0.81, 0.20)	0.2520	> 7.44	33.88 (− 0.75, 68.51)	0.0677
	≤ 2.76	− 0.21 (− 0.68, 0.25)	0.3770	≤ 4.19	1.22 (− 1.35, 3.78)	0.3623	≤ 5.08	0.14 (− 0.55, 0.82)	0.7013	≤ 7.44	− 118.09 (− 369.04, 132.86)	0.3659
IGT	> 6.48	1.31 (0.28, 2.33)	0.0235	> 5.49	2.57 (− 1.73, 6.87)	0.2591	> 3.63	− 12.94 (− 24.64, − 1.25)	0.0437	> 5.49	− 58.38 (− 97.32, − 19.44)	0.0096
	≤ 6.48	− 1.32 (− 2.67, 0.02)	0.0718	≤ 5.49	− 2.84 (− 5.52, − 0.17)	0.0532	≤ 3.63	0.05 (− 0.37, 0.47)	0.8173	≤ 5.49	5.74 (− 18.22, 29.71)	0.6448
Diabetes	> 9.71	− 0.17 (− 0.63, 0.29) 0.4725		> 3.33	− 20.05 (− 38.24, − 1.86)	0.0395	> 5.24	1.80 (− 0.86, 4.45)	0.1945	> 4.1	− 22.54 (− 57.37, 12.29)	0.2152
	≤ 9.71	1.03 (0.15, 1.91)	0.0300	≤ 3.33	0.10 (− 0.30, 0.51)	0.6208	≤ 5.24	− 0.88 (− 1.62, − 0.15)	0.0256	≤ 4.1	1.36 (− 1.63, 4.35)	0.3809

Effect: HbA1c, FPG, FPI, 0.5hPPG, HOMA-IR, ISI (the liver), ISI (the body), CIR; cause: S100A11

Adjusted: gender, age, BMI, WHR, 0.5hPPG/0.5hPPI/HbA1c/FPG/FPI

In the IGT group, there was a nonlinear correlation between serum S100A11 levels and HOMA-IR, FPI, and whole-body ISI (WBISI) after multivariate adjustment. Serum S100A11 levels lower than 6.48 ng/mL were positively correlated with HOMA-IR and FPI (a 1.31 increase in HOMA-IR and a 4.07 μU/mL increase in FPI per 1 ng/mL increase in S100A11). Serum S100A11 levels higher than 6.48 ng/mL were negatively but weakly correlated with 0.5-h PPG. Serum S100A11 levels lower than 3.63 ng/mL were negatively correlated with WBISI (a decrease of 12.94 per 1 ng/mL increase in S100A11). Serum S100A11 levels higher than 3.63 ng/mL were positively but weakly correlated with WBISI (Table 3). Multiple linear regression analysis showed a negative linear correlation between S100A11 levels and CIR. After curve-fitting, there was a significant negative nonlinear correlation between serum S100A11 levels lower than 3.63 ng/mL and CIR (a decrease of 58.38 per 1 ng/mL increase in S100A11). Serum S100A11 levels were nonlinearly correlated with HOMA-β, HbA1c, hepatic ISI, and 0.5-h PPG (Additional file 1: Figure S1); however, the changes at the inflection point were not significant (Additional file 1: Table S3).

In the group with DM, there was a nonlinear correlation between serum S100A11 levels and HOMA-IR, FPI, FPG, hepatic ISI, and HbA1c after multivariate adjustment. Furthermore, there was a positive correlation between serum S100A11 levels higher than 12.4 ng/ml, FPG, and HbA1c (a 0.88 mmol/L increase in FPG and a 0.9% increase in HbA1c per 1 ng/mL increase in S100A11). Serum S100A11 levels higher than 9.71 ng/mL were positively correlated with HOMA-IR (an increase of 1.08 per 1 ng/mL increase in S100A11). Serum S100A11 levels lower than 4.1 ng/mL were positively correlated with FPI (an increase of 8.61 μU/mL per 1 ng/mL increase in S100A11). Serum S100A11 levels lower than 3.33 ng/mL were negatively correlated with hepatic ISI (a decrease of 20.05 per 1 ng/mL increase in S100A11) (Table 3). There was a nonlinear correlation between serum S100A11 levels, HOMA-β, and 0.5-h PPI (Additional file 1: Figure S1). Nonetheless, the changes at the inflection point were not significant (Additional file 1: Table S3).

**Fig. 3:**
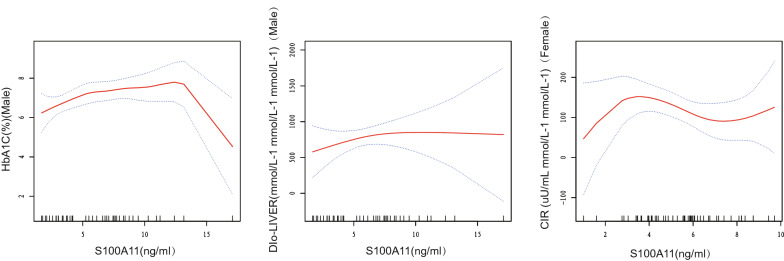
Multivariate adjusted smoothing spline plots of metabolic markers by serum S100A11, stratified by gender. Red dotted lines represent the spline plots of S100A11 and blue dotted lines represent the 95% confidence intervals of the spline plots. Adjusted for gender, age, BMI, WHR, 0.5hPPG/0.5hPPI/HbA1c/FPG/FPI

A non-linear relationship between serum S100A11 levels and glucose metabolism markers was also found by curve fitting in different gender groups (Fig. 3). In the male group, there was a nonlinear correlation between serum S100A11 levels, HbA1c, and DIo after multivariate adjustment. Serum S100A11 levels lower than 5.49 ng/mL were positively correlated with DIo (an increase of 200.77 per 1 ng/mL increase in S100A11). Serum S100A11 levels lower than 11.24 ng/mL were positively correlated with HbA1c (an increase of 0.16% per 1 ng/mL increase in S100A11) and negatively correlated with HbA1c (a decrease of 0.51% per 1 ng/mL increase in S100A11) (Table 4). In addition, there was a nonlinear correlation between serum S100A11 levels and FPG (Additional file 1: Figure S1). However, the changes at the inflection point were not significant (Additional file 1: Table S4).

**Table 4:** Threshold effect analysis of S100A11 on metabolic markers by using two-piecewise linear regression, stratified by gender

	Inflection points of HbA1c			Inflection points of DIO (the liver)			Inflection points of CIR		
	β	(95%CI)	P	β	(95%CI)	P	β	(95%CI)	P
Male	> 11.24	0.16 (0.03, 0.29)	0.0245	> 5.49	200.77 (9.58, 391.96)	0.0478	> 7.44	23.70 (− 1.02, 48.42)	0.0699
	≤ 11.24	− 0.51 (− 0.98, − 0.05)	0.0388	≤ 5.49	− 47.30 (− 143.29, 48.69)	0.3414	≤ 7.44	− 11.20 (− 37.60, 15.20)	0.4122
Female	> 5.28	0.23 (− 0.04, 0.50)	0.1076	> 3.04	676.51 (− 65.08, 1418.11)	0.0802	> 2.85	113.93 (24.32, 203.54)	0.0165
	≤ 5.28	− 0.13 (− 0.36, 0.09)	0.2393	≤ 3.04	− 51.17 (− 204.06, 101.73)	0.5151	≤ 2.85	− 14.30 (− 30.96, 2.35)	0.0992

Effect: HbA1c, DIo (the liver), CIR; cause: S100A11

Adjusted: glucose tolerance, age, BMI, WHR, 0.5hPPG/0.5hPPI/HbA1c/FPG/FPI

In the female population, there was a nonlinear correlation between serum S100A11, CIR, and 0.5-h PPI after multivariate adjustment (Fig. 2, Additional file 1: Figure S1). Nonetheless, the correlation between S100A11 and 0.5-h PPI was not significant for any change in 0.5-h PPI caused by the positive or negative correlation at the inflection point (Additional file 1: Table S4). Serum S100A11 levels lower than 2.85 ng/mL were positively correlated with CIR (an increase of 113.93 per 1 ng/mL increase in S100A11) (Table 4).

### S100A11 expression in the mouse liver

**Fig. 4:**
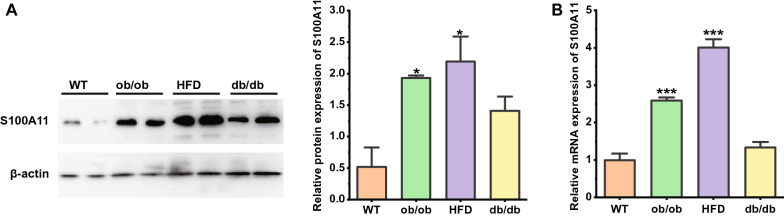
Increased S100A11 expression in the liver of obese mice. **A** Western blots and relative protein expression of total liver lysates from mice fed with HFD diet and ob/ob, db/db mice. **B** Comparison of S100A11 mRNA levels in livers of wild type and ob/ob, db/db, HFD fed mice. Data represent the means ± SEM. *P < 0.05; **P < 0.01; ***P < 0.001

We next analyzed the mRNA and protein expression of S100A11 in the liver of obese mice, diabetic mice, and mice fed a high-fat diet for 12 weeks was analyzed by qPCR and Western blotting. The results showed that S100A11 mRNA and protein levels increased in the liver of these three groups compared with normal controls (Fig. 4).

## Discussion

Hyperglycemia and insulin resistance are prominent features of type 2 DM. HbA1c, the gold standard for long-term glycemic control [14], has been shown by META analysis to be associated with an increased or decreased risk of developing many cancers in populations with or without confirmed diabetes [20, 21]. And a linear positive correlation with S100A11 was found in the human islet global genome and transcriptome [12]. S100A11, a member of the S100 family, has two calcium-binding EF-hand domains. Calcium binding to S100A11 induces conformational changes that expose a hydrophobic surface, strengthening the interaction of S100A11 with target proteins, including annexins [6, 11]. S100A11 regulates cytoskeleton dynamics and mediates cell growth, migration, and invasion [10, 22]. S100A11 is overexpressed in several cancers, including pancreatic cancer. In this respect, S100A11 expression was higher in bulk pancreatic cancer [9] tissues and intraductal papillary mucinous neoplasm than in non-neoplastic pancreatic tissue, and expression decreased with disease progression. Similarly, the incidence of pancreatic cancer was higher in diabetic patients at disease onset than in non-diabetic patients, and incidence decreased with disease progression [23, 24]. Most patients with pancreatic cancer have been found to be hyperglycemic before diagnosis, and META analysis and others support a positive correlation between HbA1c and pancreatic cancer incidence and mortality [20, 21, 23, 25, 26]. In our study, S100A11 expression increased as diabetes progressed, then whether it can be considered to play a role in the diabetic process, so we analyzed the relationship between S100A11 and related indicators in the glucose metabolic process. Fadista et al. investigated genes associated with HbA1c levels using RNA-seq and microarray and found a positive correlation between HbA1c and S100A11 levels after adjusting for gender and age [12]. In contrast, our results showed a positive linear correlation between HbA1c levels and S100A11 in the male population after adjusting for risk factors. After adjusting for all covariates, S100A11 was nonlinearly correlated with HbA1c levels in patients with IGT and in the male population, in line with the findings of Fadista et al. In addition, S100A11 was nonlinearly correlated with FPG and 0.5-h PPG levels, demonstrating the role of S100A11 levels in glucose metabolism.

Increased insulin resistance and abnormal islet cell function underlie impaired glucose metabolism. We found that S100A11 levels increased in people with IGT. Thus, we hypothesized that S100A11 affected glucose metabolism by modulating insulin action and tested this hypothesis by analyzing the relationship between S100A11 levels and insulin indices. HOMA-IR and HOMA-β are markers of insulin resistance and β-cell function [15], respectively. Oral glucose tolerance test-derived ISI [17] estimates hepatic and whole-body insulin sensitivity and CIR [100 × 30-min insulin (μU/mL)]/ [30-min glucose (mmol/L)] ×  [30-min glucose (mmol/L) – 3.89 mmol/L)] assesses β-cell function. DIo (CIR × ISI) estimates β-cell function adjusted for insulin resistance and considers the degree of insulin sensitivity [18]. Our results showed a significant positive linear correlation between S100A11 levels and HOMA-IR in the male population and a significant nonlinear correlation between S100A11 levels and metabolic markers in patients with different glucose tolerance, suggesting that S100A11 has a role in insulin resistance and islet β-cell function.

Since the liver has long been considered an important site for regulating glucose homeostasis and insulin sensitivity, we also measured S100A11 protein and mRNA expression in the livers of obese mice, diabetic mice, and mice fed a high-fat diet. As observed in humans, we found that liver S100A11 protein and mRNA were significantly increased in ob/ob, db/db, and HFD mice compared to normal controls. These liver-specific data further suggest that S100A11 may play a role in the pathogenesis of impaired glucose metabolism and insulin resistance. It suggests that we can further analyze the specific mechanism of its action on glucose and insulin metabolism through different mouse models, focusing on hepatic S100A11, and then achieve the purpose of targeting therapy and serving human clinic.

The present study analyzed the role of S100A11 in glucose and insulin metabolism. In patients with different glucose tolerance and different genders, serum S100A11 levels were not only positively correlated with HbA1c in a purely linear manner, but also had, nonlinear correlation and different positive and negative correlation effects at different thresholds. Furthermore, S100A11 was nonlinearly correlated with FPG, FPI, HOMA-IR, hepatic ISI, WBISI, and CIR in patients with IGT and DM, suggesting that S100A11 is involved in insulin resistance, pancreatic β-cell function, and hepatic and whole-body insulin sensitivity. In the male population, S100A11 levels were nonlinearly correlated with HbA1c, CIR, and DIo (derived from hepatic ISI). Moreover, in the male population, S100A11 levels could predict β-cell function after adjusting for insulin resistance and considering the degree of insulin sensitivity. These results confirm that S100A11 plays a role in the progression of DM, and its expression is closely related to glucose metabolism. Therefore, S100A11 can be used to diagnose prediabetes, and S100A11 combined with HbA1c can monitor long-term glycemic control. Nevertheless, further studies on the effects of S100A11 on glucose and insulin metabolism and islet β-cell function are needed to assess the therapeutic potential of this marker.

This study has limitations. First, we did not use statistical methods to measure the sample size, and the small sample size may lead to bias. Second, we did not analyze other metabolic markers, potentially leading to confounding bias. Third, some glucose metabolism-related indices analyzed in this study are not currently used in the clinic. Fourth, the mechanism of action of S100A11 was not investigated.

## Conclusion

This is to our knowledge the first analysis that meticulously explores the linear and nonlinear relationship of S100A11 with glucose metabolism-related indicators in different glucose tolerance populations and in different gender groups. The analysis confirmed that serum S100A11 levels were highly expressed in patients with impaired glucose tolerance, and similar results were seen in the livers of mice modeled with obesity, diabetes and high-fat diets. Significant correlations with glucose metabolism indexes were observed, suggesting a possible role of S100A11 in glucose metabolism, insulin resistance and sensitivity, and pancreatic β-cell function. However, we only analyzed the correlations, and the specific mechanism of its role in disease progression remains to be elucidated.

## Supplementary Information

**Additional file 1: Table S1.** Multivariate regression for effect of serum S100A11 level on metabolic markers in male group. Crude model: we did not adjust other covariates. Adjusted model I: We adjusted age, BMI, WHR. Adjusted model II: We adjusted AGE, BMI, WHR, HbA1c/0.5hPPI/0.5hPPG/FPG/FPI, glucose tolerance. **Table S2.** Multivariate regression for effect of serum S100A11 level on metabolic markers in female group. Crude mode I: we did not adjust other covariates. Adjusted model I: We adjusted age, BMI, WHR. Adjusted model II: We adjusted AGE, BMI, WHR, HbA1c/0.5hPPI/0.5hPPG/FPG/FPI, glucose tolerance. **Table S3.** Threshold effect analysis of S100A11 on metabolic markers by using two-piecewise linear regression, stratified by glucose tolerance. Effect: HbA1c, 0.5hPPI, 0.5hPPG, HOMA-β, ISI (the liver); cause: S100A11. Adjusted: sex, age, BMI, WHR, 0.5hPPG/0.5hPPI/HbA1c/FPG/FPI. **Table S4.** Threshold effect analysis of S100A11 on metabolic markers by using two-piecewise linear regression, stratified by sex. Effect: FPG, 0.5hPPI; cause: S100A11. Adjusted: glucose tolerance, age, BMI, WHR, 0.5hPPG/0.5hPPI/HbA1c/FPG/FPI. **Figure S1.** Multivariate adjusted smoothing spline plots of metabolic markers by serum S100A11. Red dotted lines represent the spline plots of S100A11 and blue dotted lines represent the 95% confidence intervals of the spline plots. Adjusted for sex/glucose tolerance, age, BMI, WHR, 0.5hPPG/0.5hPPI/HbA1c/FPG/FPI.

## Data Availability

Data is available through the corresponding author upon request.
