# Supplementary material for: Association between serum S100A11 levels and glucose metabolism in diabetic process
Source: Diabetol Metab Syndr. 2023 Mar 6;15:36. doi: 10.1186/s13098-023-01004-1 (PMC9987151; doi:10.1186/s13098-023-01004-1)

**Supplementary Legends:**

**Supplementary table 1.** Multivariate regression for effect of serum S100A11 level on metabolic markers in male group.

Crude model：we did not adjust other covariates.

Adjusted model Ⅰ: We adjusted age, BMI, WHR.

Adjusted model Ⅱ: We adjusted AGE, BMI, WHR, HbA1c/0.5hPPI/0.5hPPG/FPG/FPI, glucose tolerance.

**Supplementary table 2.** Multivariate regression for effect of serum S100A11 level on metabolic markers in female group.

Crude model：we did not adjust other covariates.

Adjusted model Ⅰ: We adjusted age, BMI, WHR.

Adjusted model Ⅱ: We adjusted AGE, BMI, WHR, HbA1c/0.5hPPI/0.5hPPG/FPG/FPI, glucose tolerance.

**Supplementary table 3.** Threshold effect analysis of S100A11 on metabolic markers by using two-piecewise linear regression, stratified by glucose tolerance.

Effect: HbA1c、0.5hPPI、0.5hPPG、HOMA-β、ISI (the liver); cause: S100A11.

Adjusted: sex, age, BMI, WHR, 0.5hPPG/0.5hPPI/HbA1c/FPG/FPI.

**Supplementary table 4.** Threshold effect analysis of S100A11 on metabolic markers by using two-piecewise linear regression, stratified by sex.

Effect: FPG、0.5hPPI; cause: S100A11.

Adjusted: glucose tolerance, age, BMI, WHR, 0.5hPPG/0.5hPPI/HbA1c/FPG/FPI.

**Supplementary figure 1.** Multivariate adjusted smoothing spline plots of metabolic markers by serum S100A11. Red dotted lines represent the spline plots of S100A11 and blue dotted lines represent the 95% confidence intervals of the spline plots. Adjusted for sex/glucose tolerance, age, BMI, WHR, 0.5hPPG/0.5hPPI/HbA1c/FPG/FPI.

**Supplementary table 1.** Multivariate regression for effect of serum S100A11 level on metabolic markers in male group.

|  | HbA1c（β（95%CI）P） | FPG（β（95%CI）P） | 0.5hPPG（β（95%CI）P） | DIO (the body)（β（95%CI）P） |
| --- | --- | --- | --- | --- |
| Non-adjusted ModelⅠ | 0.28 (0.10, 0.46) 0.0036 | 0.46 (0.22, 0.71) 0.0007 | 0.59 (0.27, 0.91) 0.0009 | -5.86 (-9.86, -1.86) 0.0066 |
| Adjusted ModelⅡ | 0.29 (0.10, 0.49) 0.0047 | 0.48 (0.21, 0.76) 0.0016 | 0.62 (0.27, 0.97) 0.0014 | -5.02 (-9.15, -0.88) 0.0228 |
| Adjusted Model Ⅲ | 0.07 (-0.05, 0.20) 0.2574 | 0.02 (-0.10, 0.15) 0.7011 | -0.02 (-0.19, 0.14) 0.8040 | -1.27 (-5.67, 3.13) 0.5758 |

**Supplementary table 2.** Multivariate regression for effect of serum S100A11 level on metabolic markers in female group.

|  | 0.5hPPG（β（95%CI）P） | DIO (the liver)（β（95%CI）P） | CIR（β（95%CI）P） |
| --- | --- | --- | --- |
| Non-adjusted ModelⅠ | 0.49 (-0.08, 1.06) 0.0981 | -150.28 (-297.78, -2.79) 0.0509 | -23.83 (-42.68, -4.98) 0.0164 |
| Adjusted ModelⅡ | 0.64 (0.11, 1.17) 0.0226 | -169.67 (-317.32, -22.03) 0.0286 | -25.24 (-41.76, -8.71) 0.0043 |
| Adjusted Model Ⅲ | 0.22 (-0.08, 0.52) 0.1546 | 2.63 (-142.12, 147.39) 0.9717 | -5.83 (-22.26, 10.60) 0.4901 |

**Supplementary table 3.** Threshold effect analysis of S100A11 on metabolic markers by using two-piecewise linear regression, stratified by glucose tolerance.

|  | Inflection point of HaA1c | | | Inflection point of 0.5hPPI | | |
| --- | --- | --- | --- | --- | --- | --- |
|  |  | β（95%CI）P | Log likelihood ratio |  | β（95%CI）P | Log likelihood ratio |
| Normal | ＞5.78 | 0.26 (0.04, 0.47) 0.0316 | 0.005 | ＞4.12 | 27.55 (-4.76, 59.87) 0.1088 | 0.145 |
|  | ≤5.78 | -0.43 (-0.85, -0.02) 0.0538 |  | ≤4.12 | -3.61 (-29.59, 22.37) 0.7879 |  |
| IGT | ＞8.74 | -0.11 (-0.22, 0.01) 0.1010 | 0.014 | ＞4.75 | -32.62 (-75.03, 9.79) 0.1524 | 0.08 |
|  | ≤8.74 | 3.41 (-0.03, 6.85) 0.0708 |  | ≤4.75 | 3.79 (-9.37, 16.96) 0.5806 |  |
| Diabetes | ＞12.4 | 0.11 (-0.05, 0.28) 0.1979 | 0.005 | ＞4.1 | -23.22 (-47.76, 1.32) 0.0746 | 0.035 |
|  | ≤12.4 | -0.90 (-1.63, -0.18) 0.0213 |  | ≤4.1 | 1.04 (-1.06, 3.15) 0.3399 |  |

|  | Inflection point of 0.5hPPG | | | Inflection point of HOMA-β | | | Inflection point of ISI (the liver) | | |
| --- | --- | --- | --- | --- | --- | --- | --- | --- | --- |
|  |  | β（95%CI）P | Log likelihood ratio |  | β（95%CI）P | Log likelihood ratio |  | β（95%CI）P | Log likelihood ratio |
| Normal | ＞4.19 | -0.51 (-0.98, -0.03) 0.0482 | 0.044 | ＞4.19 | -15.24 (-61.01, 30.53) 0.5206 | 0.302 | ＞4.19 | -1.06 (-4.13, 2.01) 0.5061 | 0.273 |
|  | ≤4.19 | 0.15 (-0.26, 0.55) 0.4804 |  | ≤4.19 | 16.67 (-21.59, 54.93) 0.4020 |  | ≤4.19 | 1.22 (-1.35, 3.78) 0.3623 |  |
| IGT | ＞3.63 | -10.72 (-23.94, 2.50) 0.1329 | 0.04 | ＞6.48 | 19.71 (-5.39, 44.82) 0.1433 | 0.033 | ＞5.49 | 2.57 (-1.73, 6.87) 0.2591 | 0.039 |
|  | ≤3.63 | 0.40 (-0.05, 0.85) 0.1028 |  | ≤6.48 | -25.87 (-58.78, 7.04) 0.1429 |  | ≤5.49 | -2.84 (-5.52, -0.17) 0.0532 |  |
| Diabetes | ＞3.97 | 2.36 (-1.82, 6.55) 0.2783 | 0.196 | ＞3.97 | 41.56 (-0.68, 83.79) 0.0640 | 0.031 | ＞3.33 | -20.05 (-38.24, -1.86) 0.0395 | 0.016 |
|  | ≤3.97 | -0.10 (-0.39, 0.20) 0.5325 |  | ≤3.97 | -1.37 (-4.51, 1.78) 0.4023 |  | ≤3.33 | 0.10 (-0.30, 0.51) 0.6208 |  |

**Supplementary table 4.** Threshold effect analysis of S100A11 on metabolic markers by using two-piecewise linear regression, stratified by sex.

|  | Inflection point of FPG | | | Inflection point of 0.5hPPI | | |
| --- | --- | --- | --- | --- | --- | --- |
|  |  | β（95%CI）P | Log likelihood ratio |  | β（95%CI）P | Log likelihood ratio |
| Male | ＞11.24 | -0.06 (-0.20, 0.08) 0.4148 | 0.021 | ＞2.83 | 39.63 (-8.30, 87.55) 0.1159 | 0.068 |
|  | ≤11.24 | 0.49 (0.02, 0.96) 0.0508 |  | ≤2.83 | 0.65 (-4.01, 5.30) 0.7872 |  |
| Female | ＞2.85 | 0.34 (-0.63, 1.31) 0.4940 | 0.287 | ＞4.12 | 24.24 (-3.32, 51.79) 0.0917 | 0.045 |
|  | ≤2.85 | -0.15 (-0.33, 0.02) 0.0920 |  | ≤4.12 | -5.43 (-16.18, 5.32) 0.3275 |  |

**Supplementary figure 1.**


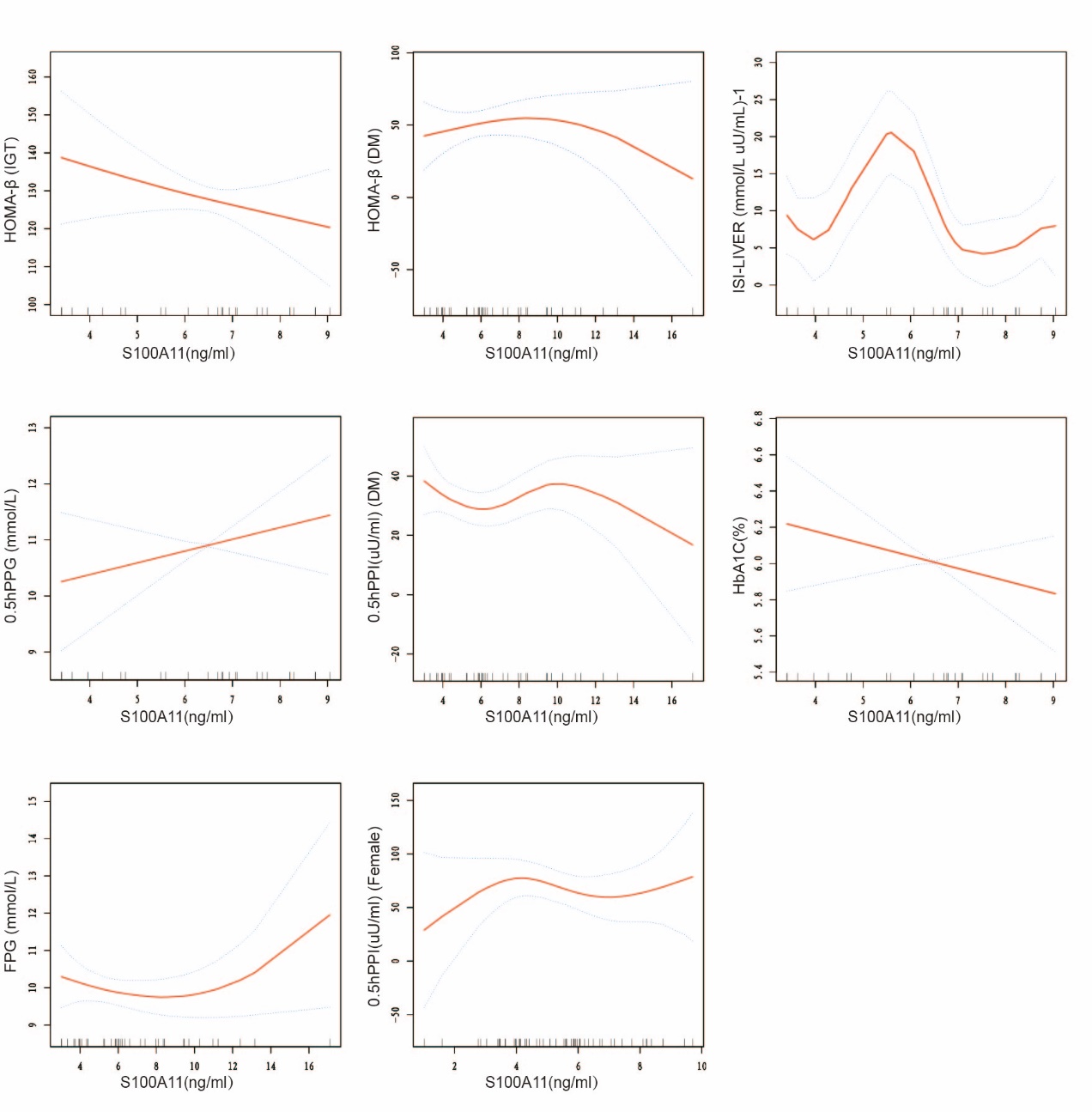

Supplement: Supplementary file 1 — Additional file 1: Table S1. Multivariate regression for effect of serum S100A11 level on metabolic markers in male group. Crude model: we did not adjust other covariates. Adjusted model I: We adjusted age, BMI, WHR. Adjusted model II: We adjusted AGE, BMI, WHR, HbA1c/0.5hPPI/0.5hPPG/FPG/FPI, glucose tolerance. Table S2. Multivariate regression for effect of serum S100A11 level on metabolic markers in female group. Crude mode I: we did not adjust other covariates. Adjusted model I: We adjusted age, BMI, WHR. Adjusted model II: We adjusted AGE, BMI, WHR, HbA1c/0.5hPPI/0.5hPPG/FPG/FPI, glucose tolerance. Table S3. Threshold effect analysis of S100A11 on metabolic markers by using two-piecewise linear regression, stratified by glucose tolerance. Effect: HbA1c, 0.5hPPI, 0.5hPPG, HOMA-β, ISI (the liver); cause: S100A11. Adjusted: sex, age, BMI, WHR, 0.5hPPG/0.5hPPI/HbA1c/FPG/FPI. Table S4. Threshold effect analysis of S100A11 on metabolic markers by using two-piecewise linear regression, stratified by sex. Effect: FPG, 0.5hPPI; cause: S100A11. Adjusted: glucose tolerance, age, BMI, WHR, 0.5hPPG/0.5hPPI/HbA1c/FPG/FPI. Figure S1. Multivariate adjusted smoothing spline plots of metabolic markers by serum S100A11. Red dotted lines represent the spline plots of S100A11 and blue dotted lines represent the 95% confidence intervals of the spline plots. Adjusted for sex/glucose tolerance, age, BMI, WHR, 0.5hPPG/0.5hPPI/HbA1c/FPG/FPI. [file 13098_2023_1004_MOESM1_ESM.docx]
